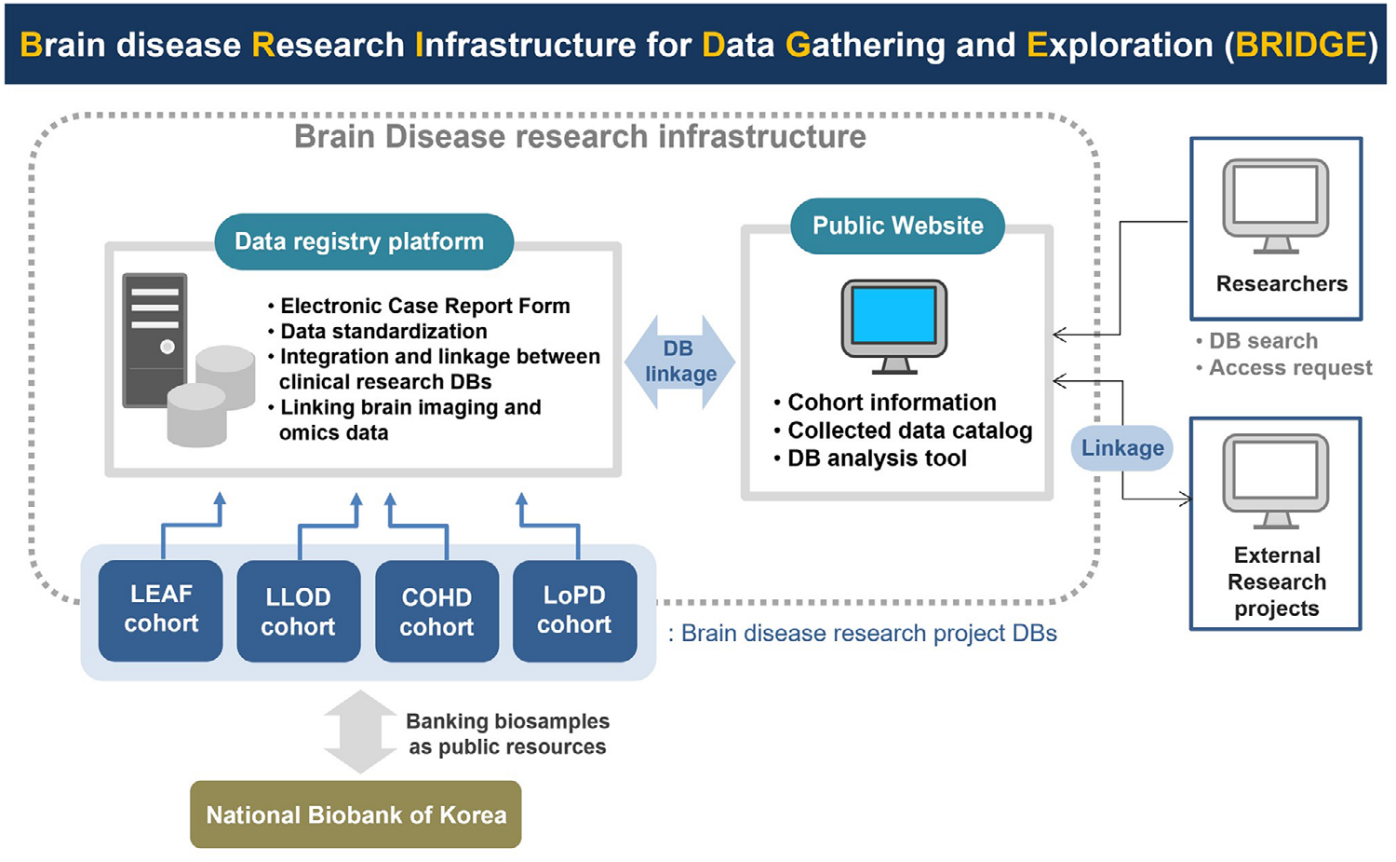# Brain disease Research Infrastructure for Data Gathering and Exploration (BRIDGE): A government‐led platform for standardized data collection, longitudinal monitoring, and sharing of Korean data sources for brain disorders

**DOI:** 10.1002/alz.094578

**Published:** 2025-01-09

**Authors:** Junwoo Seo, Sujung Oh, Eunjee Kang, Danbee Kang

**Affiliations:** ^1^ SAIHST, Sungkyunkwan University, Seoul Korea, Republic of (South); ^2^ Samsung Medical Center, Seoul Korea, Republic of (South)

## Abstract

**Background:**

Brain diseases complexity have necessitated advanced research platforms for better understanding, treatment, and prevention strategies. However, existing brain disease registries face limitations such as incomplete variable sets, lack of standardization, insufficient linkage to external databases, absence of integrated platforms for comprehensive data collection, and lack of continuity. To address these challenges, the Korea National Institute of Health initiated the Brain disease Research Infrastructure for Data Gathering and Exploration (BRIDGE), a national prospective platform designed to overcome the shortcomings of current registries.

**Method:**

The BRIDGE platform includes a Longitudinal Study of Early onset dementia And Family members (LEAF) cohort, a Longitudinal/cohort Study of Patients with Late Onset Dementia (LLOD) cohort, a Community‐based cohort study of High‐risk individuals for Dementia (COHD) cohort, and a Longitudinal Study of Patients with Parkinson’s Disease (LoPD) cohort. The standardized variables included sociodemographic variables, health behaviors, medical history, activities of daily living, behavioral, and psychological problems, cognitive function, disease‐related symptoms, quality of life (QoL), sleep, depression scale, caregiver burden, physical health, blood tests, olfactory function testing, orthostatic blood pressure changes, genetic testing, nerve conduction studies, and neuroimaging. In addition, the BRIDGE platform will1 be linked to the Korean National Health Insurance Service (K‐NHIS) database.

**Result:**

From May 2021 to December 2023, 3,505 participants were registered, with an average age of 69 years and 59.9% female. The LEAF cohort had the lowest K‐MMSE score and the highest Clinical Dementia Rating (CDR) score. The LOPD cohort had the lowest QoL scores. When compared to the age‐sex‐matched general population from Korea National Health and Nutrition Examination Survey (KNHANES), patients with brain disease were less likely to work, had lower income and had significantly lower QoL.

**Conclusion:**

By addressing gaps in data collection, standardization, and considering a wide range of impacts, the BRIDGE database offers new pathways for understanding and combating complex brain conditions. As the project progresses, it has the potential to significantly influence scientific understanding and policymaking in the field of brain health.